# Populations genetically rifting within a complex geological system: The case of strong structure and low genetic diversity in the migratory freshwater catfish, *Bagrus docmak,* in East Africa

**DOI:** 10.1002/ece3.3153

**Published:** 2017-06-30

**Authors:** Rose Komugisha Basiita, Kyall Richard Zenger, Dean Robert Jerry

**Affiliations:** ^1^ Centre for Sustainable Tropical Fisheries and Aquaculture and College of Marine and Environmental Sciences James Cook University Townsville Qld Australia; ^2^ National Agricultural Research Organization National Fisheries Resources Research Institute Aquaculture Research and Development Center Kajjansi Kampala Uganda

**Keywords:** aquaculture, *Bagrus*, genetics, Lake Victoria, microevolution

## Abstract

The complex geological history of East Africa has been a driving factor in the rapid evolution of teleost biodiversity. While there is some understanding of how macroevolutionary drivers have shaped teleost speciation in East Africa, there is a paucity of research into how the same biogeographical factors have affected microevolutionary processes within lakes and rivers. To address this deficiency, population genetic diversity, demography, and structure were investigated in a widely distributed and migratory (potamodromous) African teleost species, Ssemutundu (*Bagrus docmak*). Samples were acquired from five geographical locations in East Africa within two major drainage basins; the Albertine Rift and Lake Victoria Basin. Individuals (*N* = 175) were genotyped at 12 microsatellite loci and 93 individuals sequenced at the mitochondrial DNA control region. Results suggested populations from Lakes Edward and Victoria had undergone a severe historic bottleneck resulting in very low nucleotide diversity (π = 0.004 and 0.006, respectively) and negatively significant *Fu* values (−3.769 and −5.049; *p* < .05). Heterozygosity deficiencies and restricted effective population size (*N*_eLD_) suggested contemporary exposure of these populations to stress, consistent with reports of the species decline in the East African Region. High genetic structuring between drainages was detected at both historical (ɸ_ST_ = 0.62 for mtDNA; *p* < .001) and contemporary (microsatellite *F*_ST_ = 0.460; *p* < .001) levels. Patterns of low genetic diversity and strong population structure revealed are consistent with speciation patterns that have been linked to the complex biogeography of East Africa, suggesting that these biogeographical features have operated as both macro‐ and micro‐evolutionary forces in the formation of the East African teleost fauna.

## 1 INTRODUCTION

East African freshwater systems possess a diverse teleost fauna shaped by a complex geological history, including large‐scale tectonic movements, volcanic activity, and significant uplifting (Danley et al., 2012; Sturmbauer, Baric, Salzburger, Rüber, & Verheyen, 2001; Verheyen, Salzburger, Snoeks, & Meyer, 2003). The East African Rift (EAR) Valley, which was formed by tectonic uplift, is the major geological structure that has forged the general hydrographical network in Africa (Giddelo, Arndt, & Volckaert, 2002; Pinton, Agnèse, Paugy, & Otero, 2013) and created various freshwater habitats. For instance, major continental river systems were particularly impacted by the regional uplift, including the Nile, Congo, and Zambezi Rivers (Baker & Wohlenberg, 1971; Roberts et al., 2012). Evolutionary and geological processes such as fragmentation, hydrological connectivity, river reversal, and desiccation, among others (Danley et al., 2012; Johnson et al., 1996; Russell & Johnson, 2001), were responsible for the creation and maintenance of these habitats in which substantial numbers of aquatic taxa were isolated.

Freshwater lakes and rivers within the EAR (i.e., Lake Edward, Lake Albert, Lake George, Lake Tanganyika, Lake Malawi), and the largest tropical freshwater body, Lake Victoria (which lies outside the EAR), are habitats to one of the world's most biologically diverse aquatic faunas. For instance, prior to the introduction of the Nile perch, *Lates niloticus*, into Lake Victoria, the lake had between 350 and 600 endemic cichlids (Helfman, 2007; Turner, Seehausen, Knight, Allender, & Robinson, 2001). Geological evidence suggests a former connection of Lake Edward to Lake Victoria by late Pleistocene rivers, which were subsequently truncated by uplifting causing river reversal and a break in connectivity of lake systems (Lévêque, 1997). Presently, connectivity of these lakes is restricted to Lake Edward, which is connected to both Lakes George and Albert (via the Kazinga Channel and Semliki River, respectively). The Lake Edward‐George system provides a biogeographic confluence between the Victorian and Albertine freshwater fauna (Thieme et al., 2005). Despite this hydrological connectivity between lakes in the EAR through rivers and channels, biogeographic barriers are evident including the Semliki rapids and falls, which descends 300 m from Lake Edward to Lake Albert (Lowe‐Mcconnell, 1993, 2009), although Greenwood (1966) considers these Semliki rapids as inefficient barriers. To the right of Lake Albert are the Murchison Falls along the Victoria Nile River, another biogeographical barrier separating the Albertine rift system (includes Lakes Edward, George and Albert) from Lake Victoria. This barrier has been documented as an effective obstacle in preventing, for instance, Nile perch stocks in Lake Albert from migrating into Lakes Kyoga and Victoria (Basiita et al., 2011; Hopson, 1972).

Africa's freshwater systems are degrading at a very high rate with over 80 species listed as critically endangered, 116 species endangered and up to 103 threatened (Thieme et al., 2005). As elsewhere in Africa, the unique East African teleost faunas in both riverine and lacustrine freshwater habitats are currently under threat due to natural and anthropogenic pressures, with many species experiencing rapid population declines. Relative to other environments, biodiversity declines are at their highest in freshwater lacustrine bodies owing to the level of compartmentalization that naturally exists among these large water systems (Ricciardi & Rasmussen, 1999). The natural divides of freshwater bodies over evolutionary timescales seemingly deem them important as far as defining management units for biodiversity. Any interventions such as aquaculture developments, restocking existing water bodies, fishing zoning, and breeding grounds that are geared toward mitigating the declines resulting from natural and anthropogenic pressures need to take into account evolutionary significant units in the region.

Although numerous studies have looked at fish diversity, composition, and endemism, to understand biogeographic processes and speciation in Africa (Craig, 1992; Elmer et al., 2009; Pinton et al., 2013; Sato et al., 2003), very few fish studies have looked at genetic signatures left by biogeographical processes below the level of species (i.e., among populations). Unraveling antecedent genetic signatures among populations may not only help understand the processes that have led to their evolution and adaption in recent timescales, but more importantly, will assist with the identification of genetically divergent populations and/or evolutionally significant units that can be integrated into the formation of contemporary management plans.

*Bagrus docmak* (Ssemutundu) is a freshwater catfish with a widespread distribution in African freshwater rivers including the Nile, Chad, Niger, Volta, and Senegal. It is also found in Lake Victoria as well as the Rift Valley Lakes Edward, George, Albert, Tanganyika, Malawi, and Turkana (Aruho, Basiita, Kahwa, Bwanika, & Rutaisire, 2013; Golubtsov, Darkove, Dgebyadze, & Mina, 1995; Goossens, 2015; Greenwood, 1966; Mwanja et al., 2014). The fish species is potamodromous migrating from lakes to rivers during rainy seasons and from deep waters to shallow sandy bottoms to spawn (Chapman et al., 2012; Thieme et al., 2005). *Bagrus docmak* is an important species currently commercially fished from the wild, but also is a species with high aquaculture potential, largely because of its attractive attributes including size, taste, flesh quality, and overall commercial importance (Alhassan & Ansu‐Darko, 2011; Aruho et al., 2013; Mwanja et al., 2014). *Bagrus docmak* used to be listed as threatened by the IUCN, and the species’ overall population status where it occurs is currently unknown. Additionally, the natural populations of this species are in decline and are under threat, especially in East Africa where the species has become very rare (Aruho et al., 2013). Population declines of *B. docmak* are a result of environmental and anthropogenic pressures, such as introductions of exotic species (i.e., Nile perch, *Lates niloticus*) and habitat degradation (Chapman & Chapman, 2003; Chapman, Chapman, Kaufman, Witte, & Balirwa, 2007; Dickson, Jagwe, Longley, & Dalsgard, 2012; Hauser, Carvalho, Pitcher, & Ogutu‐Ohwayo, 1998; Kudhongania, Twongo, & Ogutu‐Ohwayo, 1992; Ogutu‐Ohwayo, 1990, 1993; Olowo & Chapman, 1999). Elucidation of *B. docmak's* population genetic structure is crucial for the species’ future management given its high conservation and commercial importance. Additionally, the species’ widespread distribution throughout Africa, along with its potamodromous migratory life history, identifies it as an ideal candidate to examine how the complex geological history of East Africa shapes evolution of the fish fauna biodiversity at below the species level.

## 2 METHODS

### 2.1 Study area

**Figure 1:**
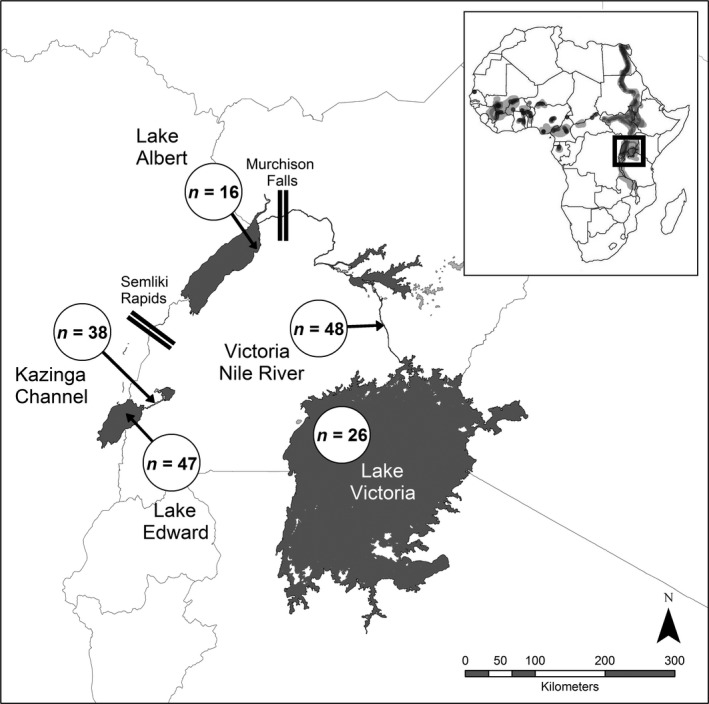
Map showing lakes and rivers (sampling locations within East Africa) and number of individuals of *Bagrus docmak* collected from each location. The inset at the top right corner denotes the distribution of *B. docmak* across Africa

Samples of *B. docmak* were collected from five water systems across the species’ distribution in central East Africa (from Lakes Albert, Edward and Victoria; and Rivers Victoria Nile and the Kazinga Channel) (Figure 1). Lakes Albert and Edward are located in the western arm of the East African Rift Valley commonly referred to as the Albertine rift, while Lake Victoria is outside.

### 2.2 Sample collection and laboratory procedures

Fin clips of *B. docmak* were obtained from commercial fishers at each of the five sampling locations (Figure 1). Samples were collected under animal ethic approval number A1824 issued at James Cook University (JCU). All fin clips were preserved in 20% dimethyl sulfoxide (DMSO) saturated with sodium chloride salt (Amos, 1991; Dawson, Raskoff, & Jacobs, 1998) and transported to the Molecular Ecology and Evolution Laboratory (MEEL) in Townsville, Australia, where they were stored at −20°C until extraction. DNA extractions and polymerase chain reaction (PCR) assays were also carried out at MEEL.

Total genomic DNA was extracted using a modified CTAB protocol (Wilson, 1990) and later using the Bioline Isolate II Genomic DNA kit. The CTAB protocol involved a digestion step using a CTAB buffer with 200 μg of Proteinase K incubated at 55°C for 2 hr, followed by a 24:1 chloroform: isoamyl alcohol purification (700 μl of chloroform–isoamyl and centrifugation at 13,200 rpm for 20 min), and an ethanol precipitation (2.5× 100% EtOH: 1:10× 5 mol/L NaAce and centrifugation at 16,000 g for 30 min, 1× 70% EtOH at 16,000 g for 20 min). Genomic DNA was re‐suspended in 25 μl of 1× TE (10 mmol/L Tris–HCl, 1 mmol/L EDTA, pH 8.0). DNA quality was estimated based on 0.8% agarose gel electrophoresis, and quantity was assessed using a ND‐1000 Spectrophotometer (Nano‐Drop^®^ Technologies). For some inhibited samples that failed to amplify during PCR, DNA was re‐extracted using a column based Bioline Isolate II Genomic DNA kit following manufacturer's protocol. Briefly the protocol involved a prelysis stage using 180 μl Lysis buffer with 25 μl of protease K incubated at 56°C. 200 μl of G‐3 lysis buffer was added and further incubated for 10 min at 70°C. 210 μl of 100% EtOH were then added to alter the buffer conditions prior to two (GW1 and GW2) buffer washes. The eluted DNA was stored at −80°C prior to downstream PCR.

DNA from 175 individuals was genotyped at 12 polymorphic microsatellite loci, Bd04, Bd18, Bd01, Bd02, Bd12, Bd09, Bd06, Bd20, Bd05, Bd03, Bd14, and Bd10 (Appendix 3) (Basiita et al. in Goossens, 2015). Forward primers were fluorescently labeled using the 5‐dye system (6‐FAM, VIC, NED, PET, and LIZ GS‐500 size standard), and all reverse primers were pigtailed (Brownstein, Carpten, & Smith, 1996) to ensure consistent amplification and minimize stuttering. A total of 20 μl PCR reactions were run on Biorad C1000 thermocycler under the following conditions: an initial denaturation at 95°C for 5 min, 6 cycles of 95°C for 30 s (denaturation)/59°C for 90 s (annealing)/72°C for 30 s (extension), 10 cycles at reduced annealing temperatures of 57, 55, and 53°C, prior to a final extension at 60°C for 30 min (Basiita et al. in Goossens, 2015). Visualization of PCR product was performed on an ABI‐3730 instrument (Applied Biosystems) using a 5‐standard dye system (6‐FAM, VIC, NED, PET, and LIZ GS‐500 size standard) at the Georgia Genomics Facility, USA. Alleles were scored using Genemarker 2.4 (Softgenetics), and checked for genotyping errors and null alleles in Microchecker 2.2.3 (Van Oosterhout, Hutchinson, Wills, & Shipley, 2004).

The Mitochondrial control region (D‐loop) was amplified in 93 individuals from the five locations. Initially, catfish oligonucleotide primers, MT16498H and L19 (Chenoweth & Hughes, 1997) were used to amplify the D‐loop mitochondrion region to obtain *B. docmak* sequences that were then used to design more robust species‐specific primer pairs. Primers specific to *Bagrus* spp. were designed using a free online software, primer3 (Rozen & Skaletsky, 2000). A forward (BagDF2)–TTGAGGGTTGGTGGTTTCTT and reverse (BagDR2)–AAACTATTTTCTGTAAATGCATAAT) primer pair were designed and tested for specificity in *B. docmak* via PCR. A total of 25 μl reaction volume was used; 2.5 μl 10× buffer, MgCl_2_ 1.5 mmol/L, dNTPs 0.2 mmol/L, *B. docmak* control region primers; BagDF2 and BagDR2 (at a final concentration of 0.2 μmol/L for each primer) and 1 μl DNA (5 ng/μl); PCR cycling conditions comprising of an initial denaturation of 94°C for 5 min, then 30 cycles of 94°C 30 s (denaturation)/50°C for 30 s (annealing)/72°C for 30 s and a final extension of 72°C for 5 min. Visualization for amplification was achieved via electrophoresis using a 1.5% agarose gel. PCR product was cleaned with Sephadex G‐50 (GE Healthcare, UK) columns prior to sending approximately 5 ng of each sample for Sanger sequencing at the Australian Genome Research Facility (AGRF), in Brisbane–Australia. Sequencing was performed in both forward and reverse directions using the designed *B. docmak* specific primers.

### 2.3 Mitochondrial DNA sequence editing and alignment

Individual D‐loop sequences were aligned and consensus sequences generated (Bast, 2013) in GENEIOUS 8.02 (Biomatters Ltd). Following generation and alignment of all consensus sequences, a 400 bp size region was trimmed and exported to MEGA 5.2 (Tamura et al., 2011). The best substitution model for analyzing sequence divergence and population structure among the different populations was selected as T92+G (Nei & Kumar, 2000; Tamura et al., 2011) using MEGA 5.2 according to Schwarz (1978).

### 2.4 Genetic diversity: Descriptive statistics

Analyzes to determine the number of alleles, observed and expected heterozygosities (*H*_o_ and *H*_e_ respectively), inbreeding coefficient (*F*_is_) and conformation to Hardy–Weinberg Equilibrium (HWE) at 12 microsatellites loci were performed in GenAlex (Peakall & Smouse, 2012) and Arlequin 3.5 (Excoffier, Lischer, & Schneider, 2010). All significance levels were corrected for multiple tests using a two‐step false discovery rate (FDR) correction (Benjamini & Hochberg, 1995; Benjamini, Krieger, & Yekutieli, 2006) set at a maximum of 0.001. The *F*_is_ was used as a measure of inbreeding and/or population subdivision. Additionally, the average level of relatedness was calculated within each population using ML‐Relate (Kalinowski, Wagner, & Taper, 2006). Input files were converted for use between programs using the free software, PGDspider 2.0.5 (Lischer & Excoffier, 2012). All microsatellite loci were polymorphic in all populations sampled, except for locus BD18 that was monomorphic in the Victoria Nile River and BD20 in the Lake Victoria populations. Micro‐checker 2.2.3 was used to detect genotyping errors, the presence of null alleles and allelic dropouts (Morin et al., 2009; Van Oosterhout et al., 2004). Diversity indices calculated for the mitochondrial data included; haplotype number (*n*), haplotype diversity (Hd), and nucleotide diversity (π). These parameters were used as a measure of genetic divergence at the mitochondrial D‐loop region within and among the sampled populations. All computations were implemented in DnaSP (Librado & Rozas, 2009) and Arlequin 3.5 (Excoffier et al., 2010).

### 2.5 Demographic history

Microsatellite allele frequencies were used to assess the recent demographic history of *B. docmak*. All three microsatellite mutation models (SMM, IAM and TPM 7:3 ratio) were used to test for signatures of population reductions for each of the five populations using Bottleneck 1.2.02 software (Cornuet & Luikart, 1996; Piry, Luikart, & Cornuet, 1999; Selkoe & Toonen, 2006).

Historical demography was investigated at the mitochondrial D‐loop by estimating *Fu's F* statistic for each of the five locations (Fu, 1997). Neutrality tests to estimate *Fu's F* statistic were carried out in Arlequin (Excoffier et al., 2010). Furthermore, mismatch analysis distribution was performed for the demographic analysis in which pairwise difference distributions and the frequency of segregating sites were analyzed in DnaSP (Librado & Rozas, 2009; Rozas, Sánchez‐Delbarrio, Messeguer, & Rozas, 2003).

### 2.6 Population structure

Genetic population structure was investigated at the D‐loop region of the mtDNA and at 12 polymorphic microsatellite loci. Using both datasets, analysis of molecular variance (AMOVA) and pairwise comparisons between locations were completed in Arlequin 3.5 (Excoffier et al., 2010). For microsatellite data, the AMOVA was based on allelic frequencies, and for mtDNA sequence data the AMOVA was based on a genetic distance matrix of pairwise differences between pairs of populations. Significance was estimated at 10,000 permutations.

Furthermore, using mitochondrial sequence data organized in DnaSP (Librado & Rozas, 2009) and exported to Network 4.611 (Flexus Technology, as reported by Bandelt et al., 1995), the population genetic structure and geographical distribution of haplotypes were visualized. Calculation and drawing of a minimum spanning network based on haplotype distribution in sampled individuals from all the five populations were completed in Network4.611 Flexus Technology.

Bayesian clustering analysis conducted in the program, STRUCTURE 2.2 (Earl & von Holdt, 2012), was used to assign individuals from the five locations into distinct genetic clusters. The analysis was based on microsatellite data at 12 polymorphic loci, and runs were conducted on putative populations (*K*) set from 1 to 10 iterations with 10,000 burn‐ins followed by 100,000 Markov‐Chain Monte Carlo (MCMC) steps for each run. Potential clusters were determined based on an MCMC approach, both with and without a priori definition of structure, and also assuming independent frequencies of alleles. Using the web‐based Structure Harvester software (Earl & von Holdt, 2012), the best *K* value was then selected. Additionally a multivariate method, the Discriminant Analysis of Principal components (DAPC), was performed in the R package adegenet v1.4.2 (Jombart, 2008), to explore a finer scale structure of the populations based on a two‐step procedure. Firstly, the genetic data are transformed using a Principal Component Analysis (PCA), and then clusters are identified by Discriminant Analysis (DA) without assuming panmixia (Jombart, 2008; Jombart, Devillard, & Balloux, 2010).

## 3 RESULTS

### 3.1 Genetic diversity

Mitochondrial DNA D‐loop variation among 93 *B. docmak* individuals from the five locations revealed high haplotype diversity, Hd, with a narrow range across populations (Hd range of 0.698 for Kazinga Channel to 0.857 for Lake Albert). The nucleotide diversity was highest in individuals from Lake Albert and the Victoria Nile river, both with π = 0.010, and least in Lakes Edward and Victoria, π = 0.004 and 0.006, respectively (Table 1). Overall there were 25 distinct haplotypes, with 31 polymorphic sites and a total of 33 mutations (Appendices 1 and 2). Haplotype sequences were deposited into GenBank with accession numbers MF118537 to MF118561.

**Table 1:** Microsatellite and mitochondrial DNA diversity indices for *Bagrus docmak* from five freshwater systems in East Africa

Location	Microsatellite data					Mitochondrial data			
	*N*	*N*_a_ (range)	*H*_o_	*H*_e_	*N*_eLD_ (95% CI)	*N*	π	Hd	*Fu's*
Lake Victoria^a^	26	3.25 (1–6)	0.199 (0.00–0.615)	0.213 (0.000– 0.706)	18.2 (6.7–127.4)	13	0.006	0.846	−3.769*^*^
Victoria Nile^a^	47	3.33 (1–5)	0.225 (0.00–0.391)	0.238 (0.000–0.48)	58.2 (22.9–6545)	18	0.010	0.750	−1.461
Lake Albert	16	4.67 (2–7)	0.552 (0.333–0.688)	0.60 (0.426–0.781)	∞ (111.6–∞)	16	0.010	0.857	−1.606
Lake Edward	48	4.42 (2–6)	0.385 (0.126–0.542)	0.381 (0.119–0.556)	31.3 (18.8–60.3)	21	0.004	0.732	−5.049**^*^
Kazinga Channel	38	4.58 (2–6)	0.382 (0.147–0.579)	0.38 (0.190–0.586)	978.5 (71.1–∞)	29	0.009	0.698	−6.033*^*^

Values for microsatellite statistics are means over all loci (range) for each location: *N*, sample size; *N*_a_, mean number of alleles; *H*_o_, mean observed heterozygosity; *H*_e_, mean expected heterozygosity; *N*_eLD_, effective population size as estimated by linkage disequilibrium method (at 95% confidence interval); π, nucleotide diversity; and Hd, haplotype diversity.

Populations marked with superscript a exhibited monomorphism at one of the twelve loci investigated. Asterisks on *Fu's* statistic denote the level of significance.

All populations were in Hardy–Weinberg equilibrium, and there was no detection of large allelic dropouts. Null alleles were not detected in the Lake Victoria population; however, they were suggested at locus BD02 for individuals sampled from the Victoria Nile River, BD01 in the Lake Albert and Lake Edward populations, and BD20 in the Kazinga Channel population. There was no locus that consistently exhibited null alleles, and as such all microsatellite loci were polymorphic across populations with number of alleles up to seven alleles per locus and overall mean allelic diversity, mean *N*_a_ ± *SE* = 4.05 ± 0.192 (Table 1). The allelic diversity was highest in Lake Albert (mean *N*_a_ = 4.67 ± 0.497) and lowest in the Lake Victoria population (mean *N*_a_ = 3.25 ± 0.494). Similarly observed and expected heterozygosities were highest in the Lake Albert population (mean *H*_o_ of 0.55 ± 0.034 and *H*_e_ 0.66 ± 0.031) and lowest in the Lake Victoria population (mean *H*_o_ = 0.199 ± 0.046 and *H*_e_ = 0.213 ± 0.053) (Table 1). Bottleneck analysis under the SMM and TPM models revealed significant heterozygosity deficiencies (with *p* values <.005) for *B. docmak* populations from Lake Victoria and Edward. These two populations additionally displayed an L‐shaped allelic distribution characteristic of populations undergoing expansion following contractions (data not shown). The effective population sizes, *N*_eLD_, for Lake Victoria, Lake Edward and the Nile River were finitely restricted (Table 1). On the contrary, the results showed that the sampled populations were mating randomly with the *F*_is_ indices generally low and showing no significant deviation from zero (*p* > .05), with the exception of Lake Victoria having the highest *F*_is_ of 0.1101 (*p* = .054), but still statistically insignificant (Table 2). Overall there was low level of relatedness in the sampled individuals from all populations (Table 2).

**Table 2:** Genetic population bottleneck tests including inbreeding coefficient *F*_IS_ and relatedness

Population	*F*_IS_ (*p* value)	Bottleneck test: IAM	Bottleneck test: SMM	Bottleneck test TPM	Percentage un relatedness
Lake Victoria	0.1101 (.054)	.0068	.0005	.0015	79.69
Victoria Nile River	0.0729 (.093)	.0068	.0005	.0010	72.34
Lake Albert	0.0570 (.195)	.3394	.9097	.6773	90.00
Lake Edward	−0.0567 (.916)	.2036	.0017	.0425	75.93
Kazinga Channel	0.0171 (.365)	.0640	.0012	.0024	80.80

The bottleneck probabilities reported for IAM, SMM, and TPM models above were Wilcoxon probability 2‐tail tests for heterozygosity deficiency and excess as implemented in the program Bottleneck (Cornuet & Luikart 1996; Piry et al. 1999).

### 3.2 Demographic history

Mismatch distribution analyzes showed varied demographic histories for *B. docmak* from the five locations. *Fu's F* statistics were negative in all populations and highly significant for *B. docmak* individuals from Lakes Victoria, Edward, and the Kazinga channel (see Table 1).

Overall, all populations sampled exhibited heterozygosity deficiencies except for the Lake Albert population that did not show significant deviations from a stable population (*p* > .37, SMM and *p* > .05, under the IAM model). The Lake Albert population exhibited characteristics of a stable population with three loci showing heterozygosity deficiency and nine loci with heterozygosity excess. Contrary to Lake Albert, results indicated that the Lake Victoria population exhibited a normal L‐shaped curve characteristic of a young and expanding population. This is further confirmed by a significantly high heterozygosity deficiency at 10 loci (*p* < .001 under the SMM model). Additionally, relative to all populations, Lake Victoria displayed the highest *F*_IS_ index of 0.11 (Table 2), although it was not significant (*p* > .05).

Similarly, using the linkage disequilibrium method for estimating the effective population size (*N*_eLD_) in *Ne*Estimator, Lake Albert showed an infinite *N*_eLD_ with infinite number of breeders, followed by the Kazinga channel which also exhibited a relatively high Ne (Ne = 998, range 71.1–∞). Populations from Lake Victoria and Lake Edward had restricted effective population sizes, *N*_eLD_ of 18.2 (6.7–127.4) and 31 (18.8–60), respectively (Table 1). The *N*_eLD_ estimates are consistent with results from the bottleneck test, as well as the mitochondrial results that showed high nucleotide diversities for populations of Lake Albert and relatively low nucleotide diversity for populations from Lake Victoria and Lake Edward (Table 1).

### 3.3 Population structure

MtDNA D‐loop analysis for *B. docmak* populations revealed highly significant structuring between populations across the Albertine Rift valley and the Lake Victoria basin populations (ɸ_ST_ = 0.62; *p* < .001). Pairwise ɸ_ST_ between populations across the two regions ranged from 0.628 to 0.820 (*p* < .001) which were up to sevenfold higher in comparison to the within‐region population ɸ_ST_ pairs (range of 0.013–0.097). Moderate‐to‐weak structuring also existed between populations within basins, especially for water systems that were geographically proximate, or directly connected with no major biogeographical barriers. For instance, pairwise ɸ_ST_ values between “Kazinga Channel–Lake Albert” and “Lake Edward–Lake Albert” showed moderately low ɸ_ST_ of 0.046 (*p* < .05) and 0.0968 (*p* < .001), respectively (Table 3). In both cases, there was relatively weak genetic structuring among these populations situated within the Albertine Rift valley. There was no significant structuring between Lake Edward and the Kazinga channel populations (*p* > .05), which is not surprising given their direct geographical connectivity. Similarly, there was weak and insignificant structuring (*p* > .05) between the Victoria Nile River samples and the Lake Victoria population.

**Table 3:** Comparison of population pairwise ɸ_ST_ based on the mitochondrial DNA data with ɸ_ST_ values below diagonal and corresponding *p* values (10,000 permutations) above diagonal

Population	Lake Victoria	Victoria Nile River	Lake Albert	Lake Edward	Kazinga Channel
Lake Victoria	–	.369	.0000	.000	.000
Victoria Nile River	.013	–	.0000	.000	.000
Lake Albert	.716	.628	–	.002	.033
Lake Edward	.820	.740	.097	–	.125
Kazinga Channel	.727	.659	.046	.0162	–

Overall fixation index, ɸ_ST_ was 0.62 at *p* < .001.

**Figure 2:**
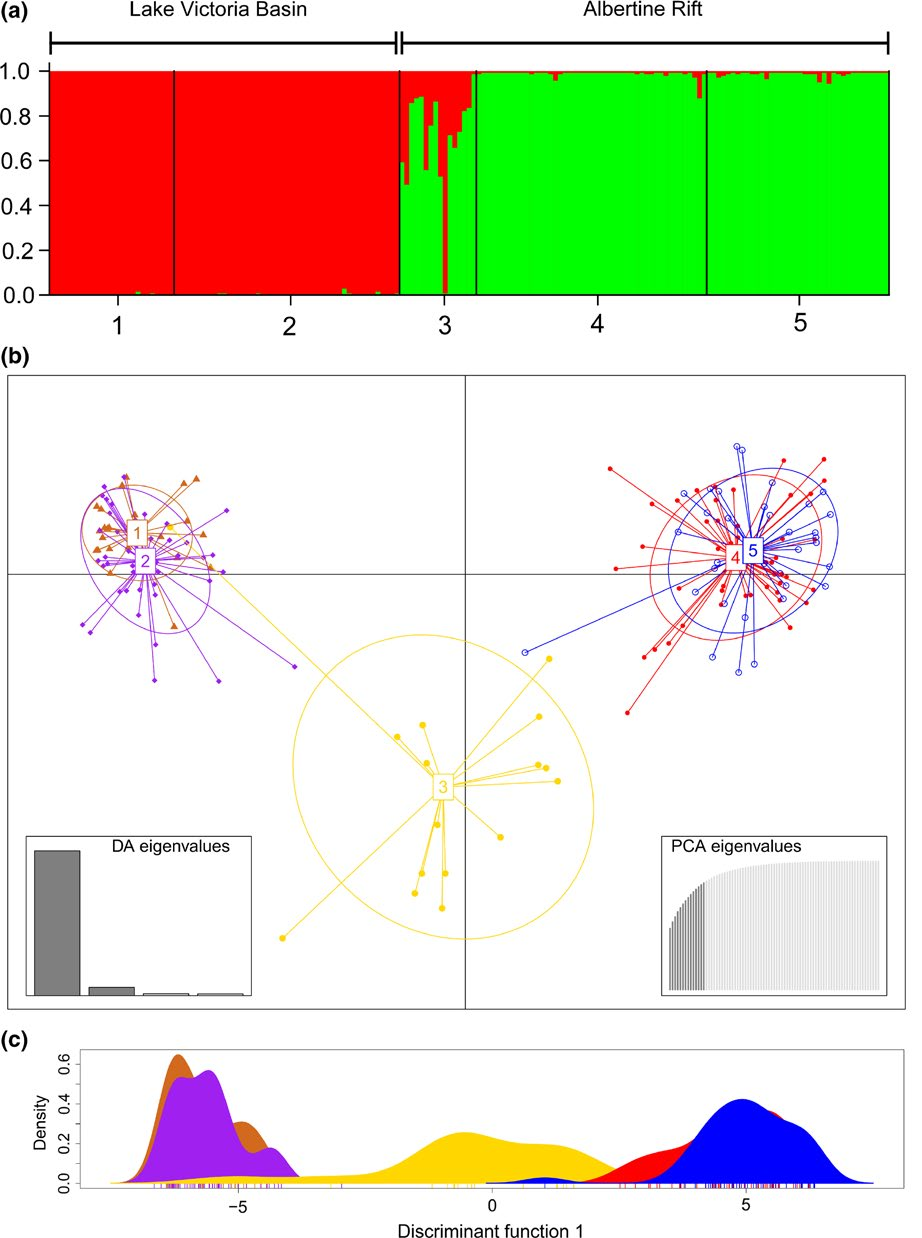
(a) STURCTURE bar plot of *Bagrus docmak* populations depicting two genetic clusters (*K* = 2) as per the genome ancestry assignment revealed. *X* axis represents individuals from the five locations (1‐Lake Victoria, 2‐Victoria Nile River, 3‐Lake Albert, 4‐Lake Edward, and 5‐Kazinga channel) assigned to two major stocks/populations; Victoria basin populations (red) and Albertine Rift populations (Green). (b) Scatterplot showing Discriminant Analysis of Principal Components (DACP) for 175 individuals of *B. docmak* in adegenet, an R package. The colors represent populations with respective number of individuals sampled; Yellow–Lake Albert with 16 individuals, Red–Kazinga Channel with 38 individuals, Gray–Nile River with 47 individuals, Orange–Lake Edward with 48 individuals, and Blue–Lake Victoria with 26 individuals. Thus, each color dot represents an individual with discrete clusters surrounded by 95% confidence interval inertia ellipses. (c) DAPC density plot based on the most variable component

Results of population structure based on microsatellite analysis mirrored the mitochondrial DNA ɸ_ST_ patterns. Here, population pairwise *F*_ST_ values were in the range of 0.014–0.549, with significant structuring between populations across the Albertine (Lakes Edward and Albert and the Kazinga Channel) and Lake Victoria basin (Lake Victoria and Victoria Nile river populations (Table 4). Additionally there was weak, but significant *F*_*ST*_ between Lake Victoria and the Nile river populations, *F*_ST_ = 0.047 (*p* < .001). Bayesian STRUCTURE analysis and DAPC multivariate analyzes also showed a similar trend with varying degrees of resolution (Figure 2a–c).

**Table 4:** Pairwise *F*_ST_ among *Bagrus docmak* populations from East Africa using microsatellite data. Below diagonal, *F*_ST_ values, above diagonal significance level corrected for FDR at .001

Population	Lake Victoria	Victoria Nile	Lake Albert	Lake Edward	Kazinga Channel
Lake Victoria	–	.001	.000	.000	.000
Victoria Nile	.047	–	.000	.000	.000
Lake Albert	.355	.379	–	.000	.000
Lake Edward	.515	.526	.150	–	.029
Kazinga Channel	.535	.549	.157	.014	–

Bayesian clustering revealed two major distinct genetic clusters based on the highest probability likelihood achieved of *K* = 2 when evaluating all populations together. One genetic cluster predominantly comprised Lake Victoria basin populations (i.e., from the Nile River and Lake Victoria) and the second genetic cluster contained populations from the Albertine Rift Valley (i.e., Lake Edward and the Kazinga Channel). However, individuals from Lake Albert comprised both genealogies from the Albertine rift and Lake Victoria basin (Figure 2a) when partitioned at this level of *K* = 2. When evaluating with DAPC, results revealed three distinct clusters (Figure 2b,c), grouping individuals from Lake Albert as a discrete population between the other two major clusters. Lake Edward and the Kazinga channel grouped into another cluster and equally the Lake Victoria and Nile River individuals into a third distinctive group. These hierarchical clustering patterns are consistent with the level of population divergence observed in the pairwise *F*_ST_ comparison data (Table 4).

**Figure 3:**
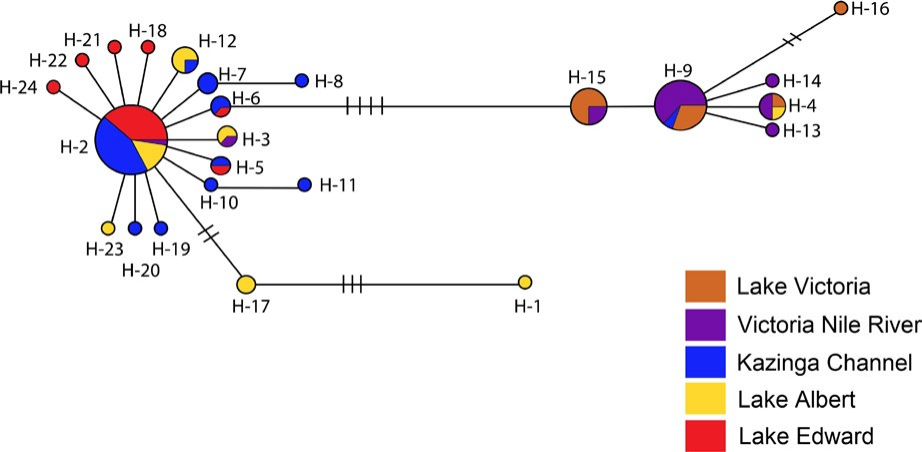
Haplotype network for *Bagrus docmak* from five geographical locations as drawn in Network 4.6.1. Each circle denotes a single haplotype whose size is proportional to the frequency of the haplotype. The colors represent the geographical source of the haplotype. Each branch indicates a single mutational event except where indicated by lines that correspond to the total number of mutations

The haplotype network based on mtDNA data for *B. docmak* was characterized by two centrally shared haplotypes, H‐2 and H‐9, (one from each of the two regions separated by up to four mutational events; the Albertine rift and the Lake Victoria basin) and 23 other smaller haplotypes that were branching out in the periphery. The most common haplotype was shared by 33 individuals restricted to the Albertine rift (Lake Edward, Lake Albert and the Kazinga channel) and representing up to 35% of the all sampled individuals. The second largest haplotype had 16 individuals restricted to the Nile River and Lake Victoria. From these two major haplotypes span other smaller haplotypes (Figure 3). These results suggest the presence of two fairly distinct genetic groups spanning the five geographical locations sampled.

## 4 DISCUSSION

### 4.1 Genetic diversity, population structure, and connectivity of *Bagrus docmak*

The complex geological history of East Africa has acted as a biogeographical evolutionary driver of the diverse freshwater fish species fauna seen in the region (Danley et al., 2012; Sturmbauer et al., 2001; Verheyen et al., 2003). Similarly, genetic analyzes of *B. docmak* populations from lacustrine and riverine habitats of the EAR show evidence for vicariance leading to genetic divergence below the species level. Both mtDNA and nuclear markers highlight the presence of significant genetic population structure among *B. docmak* populations within and outside the EAR (*F*_ST_ of 0.46 for microsatellite and ɸ_ST_ of 0.62 for mitochondrial DNA data, *p* value <.001), with two major genetic stocks identified: one within the Albertine rift and the other within the Lake Victoria basin. Mitochondrial DNA analyzes show that the separation of stocks has an ancient origin with a high degree of haplotype sorting evident, with just two major haplotypes and restricted mutations of up to four base pair changes between these two major haplotypes. The near complete haplotype sorting and reduction in diversity suggests a severe vicariant and bottlenecking event among catfish populations (Figure 3). The split lineage and highly truncated haplotype sharing between Lake Edward and Lake Victoria individuals, emphasizes, the divergence of these two lakes situated in the Albertine rift valley and Lake Victoria basin drainages, respectively. The divergence of populations of this potamodromous freshwater catfish in the EAR lakes and Lake Victoria is consistent with high degree of endemism and radiations among other African cichlids within the Great Lakes Region and the suckermouth catfish species *Chiloglanis anoterus* of the African Highveld (Johnson, Kelts, & Odada, 2000; Johnson et al., 1996; Morris et al., 2016; Russell & Johnson, 2001). Although there is hardly any information on genetic variation among other bagrid catfishes in African freshwater systems for direct comparison, the divergence of populations in the *B. docmak* was found to be higher than what has been reported in another Asian freshwater bagrid catfish, *Leiocassis longirostris* (Yang, Xiao, Yu, & Xu, 2012).

This study also highlighted evidence for admixture among Lake Albert *B. docmak* (Figure 2a). This admixture may be indicative of limited connectivity between Lakes Victoria and Albert through the Victoria Nile and is consistent with historical exchanges of fauna to have occurred previously following geomorphological events, especially in the Nile system and its associated basins (Stewart, 2009). Murchison Falls likely acts as a one way biogeographical barrier inhibiting fish stocks moving upstream from Lake Albert into Lake Victoria; however, it may not act as a barrier to fish moving downstream from Lake Victoria to Lake Albert. The biogeographical separation of Lake Albert from Lake Victoria by the Murchison Falls has been well documented, and it is this barrier that prevented the Nile perch from crossing upstream from Lake Albert into Lakes Kyoga and Victoria (Hopson, 1972).

Despite similar geological history of formation by tectonic movements and their connectivity through the Semliki River, moderate, but significant structuring was detected between populations of Lake Edward and Lake Albert based on both mitochondrial and microsatellite data. The divergence of *B. docmak* populations between these two lakes is possibly a result of the biogeographical barrier of the Semliki rapids which may restrict levels of gene flow between these two lakes (Thieme et al., 2005). This observed genetic structuring pattern is consistent with earlier species composition studies, where unique fish fauna have been identified in Lake Albert, but not in Lake Edward (Devaere, Jansen, Adriaens, & Weekers, 2007).

Correspondently, the weak and insignificant *F*_ST_ values obtained between lake–river systems (i.e., Lake Edward–Kazinga Channel River and Lake Victoria–Victoria Nile River) is consistent with the direct connection of these lake–river systems which have no known biogeographic barriers. In addition, *B. docmak* being potamodromous (Chapman et al., 2012; Manyala, Bolo, Onyango, & Rambiri, 2005), implies that gene flow between lakes and river systems will be enhanced where there are no barriers, but inhibition of gene flow will be accelerated in the presence of geological features that interrupt migration and consequently restrict gene flow. Connectivity would foster the exchange of genes of populations between the lacustrine and riverine habitats.

### 4.2 Historical and contemporary genetic signatures

Understanding biogeography using genetics is important to determine patterns influencing distribution of geographically distant populations. Evidence of sequence and haplotype divergence revealed that *B. docmak* populations (Lake Victoria, Lake Edward and the Kazinga Channel) underwent severe contractions as a result of biogeographical influences within the East African region. Generally the mitochondrial haplotypes branches were very short showing that the divergences were fairly recent with just up to four mutations between the two major lineages. It also indicates a short evolutionary time since the common ancestor. Additional evidence of low diversity in the mitochondrial data reveals that the populations from Lakes Victoria and Edward have been restricted to a very small size over a few thousands of years. High haplotype diversity coupled with low nucleotide diversity as was observed in the Lake Victoria and Edward *B. docmak* populations (Table 1), is a pattern consistent with one which has been documented in another catfish species, *L. longirostris* (Yang et al., 2012). This pattern has been associated with recent population expansions following population bottlenecks (Grant & Bowen, 1998; Yang et al., 2012). Evidence of a historical bottleneck and a possible recent colonization was also confirmed by the negative and significant *Fu's* statistic obtained in the Lake Victoria, Lake Edward, and Kazinga channel populations (Fu, 1997). The results are consistent with reports of mass extinctions in these lakes and recolonization events particularly in Lakes Edward, George, and Victoria (Beadle, 1974; Thieme et al., 2005). It should be noted that long droughts characterized the East African region during the late Pleistocene coupled with the tectonic uplifts that were responsible for drying up and river reversal between the lacustrine environments (Day et al., 2013). Additional evidence to the Pleistocene events of river reversal is the complete desiccation of Lake Victoria following a long drought period (Johnson et al., 1996). *B. docmak* being potamodromous was most likely susceptible given its migration patterns between the riverine and lacustrine habits.

Corroborative evidence was obtained using the microsatellite data in which restricted *N*_eLD_ estimates were recorded in addition to significant heterozygosity deficiencies in all the populations except for Lake Albert. The low *N*_eLD_ (Table 1), relatively high inbreeding coefficient, and significant heterozygosity deficiencies in the Lake Victoria and Edward populations, further indicate that these populations are constrained and have not had enough time to recover from the severe historic bottleneck. As pointed out in earlier studies the population in Lake Victoria is additionally constrained by anthropogenic pressures such as heavy fishing and exotic species introductions (like Nile perch) that have been clearly documented in the region (Hauser et al., 1998; Kudhongania et al., 1992; Ogutu‐Ohwayo, 1990, 1993). Evidence highlights that *B. docmak* was formally a dominant and higher‐order carnivorous fish in the Lake Victoria basin, but it currently faces direct competition for prey and is also preyed upon by the introduced Nile perch (Frans Witte, 1997).

Furthermore our study reveals a recent expansion amidst high, but insignificant *F*_IS_, thus rejecting the hypothesis of possible inbreeding. It is, therefore, predicted that the large surface area of Lake Victoria (68,000 km^2^) provides an advantage of a large habitat in which the species will survive for a while if proper management strategies are put in place. Conversely, Lake Edward which is a small (2,235 km^2^) EAR lake, has been considered hydrologically and chemically sensitive to climatic changes as it lies in the intersection of the Indian and Atlantic air masses (Russell & Johnson, 2001). The truncated gene flow between Lakes Edward, Victoria, and Albert due to geological and biogeographic barriers largely exposes Lake Edward populations to risk, especially in the event where they are exposed to stochastic events (such as disease outbreaks) and anthropogenic pressures (including heavy fishing and habitat degradation).

The exception of Lake Albert and the Victoria Nile River in showing negative, but nonsignificant neutrality tests, reveals the possibility of a long‐term demographic stability. The microsatellite data confirms the stability of the Lake Albert system following a balanced heterozygosity consistent with a stable population as assessed by the bottleneck test. Furthermore, the Lake Albert population was characterized by an infinite *N*_eLD_ estimate coupled with the highest level of heterozygosity and number of private alleles. The evidence of stability shown in the Lake Albert population suggests the presence of undisturbed habitats within this lake. Hence, it is not surprising that Lake Albert has also been referred to as an abyss with deep waters compared to the shallow Lake Victoria (Mwanja et al., 2014).

The evidence for genetic stability suggested in the Lake Albert population in the current study does not in any way warrant populations of *B. docmak* from this particular lake to be neglected for conservation; however, the findings do provide a baseline on the genetic status of the species following historical processes (Johnson et al., 1996, 2000; Russell & Johnson, 2001). The current anthropogenic pressures such as aquaculture establishments (Dickson et al., 2012) on the lake, as well as proposed oil extraction within the Albertine region (Kathman & Shannon, 2011; Vokes, 2012), could potentially accelerate habitat destruction and loss of genetic biodiversity. Therefore, a genetic baseline is needed to monitor and design appropriate management measures for the lake and the region at large.

Information provided by the current study will facilitate the comprehensive management of *B. docmak* and related taxa for sustainable harvest in the wild and/or culture under captive conditions. The strategy should consider river and lake regulations together with their associated developments, such as, damming, hydropower generation, fishing, transport, among others. Replenishment of fish stocks through restocking and aquaculture need to take into account the current and historic genetic diversity and population structure of the identified stocks. These stocks potentially translate into majorly two evolutionary significant units (evidence from mitochondrial data) and two management units (based on STRUCTURE analysis). These findings are important in developing appropriate conservation and management strategies which depend majorly on our ability to correctly assign genetically distinct populations (Latch, Dharmarajan, Glaubitz, & Rhodes, 2006).

## 5 CONCLUSION

The low genetic diversity and strong population structure patterns revealed in *B. docmak* are consistent with patterns observed in other freshwater fish species that have been linked to the complex biogeography of East Africa (Danley et al., 2012). The results in the current study support the hypothesis of Danley et al. (2012) and Pinton et al. (2013), who postulate that a linkage of paleohydrological changes in a geological context has been a major cause of diversification of the freshwater teleost fauna in East Africa. Particularly, the East African Rift system and associated historical events appear as strong drivers of freshwater diversification and evolution. In the case of *B. docmak*, both population fragmentation and reductions in population size due to the complex geological and climate variability in the EAR have resulted in significant genetic structure among populations.

Strategically, the management of aquatic fauna in the East African region should initially take into account the two major management units identified, that is the Albertine rift valley region and the Lake Victoria basin. However, further research is required for the Lake Albert population which was singled out as a discrete group at a finer scale with DAPC analysis to make a total of apparently three management units comprising of Lake Edward and Kazinga Channel cluster, Lake Victoria–Nile River cluster and Lake Albert.

## CONFLICT OF INTEREST

None declared.

## APPENDIX 1

### List of haplotypes for *Bagrus docmak*

**Table:**

31 Polymorphic sites																																Haplotype distribution and frequency					Totals
																																VIC	VIC_NILE	ALB	EDW	KAZ	
	1	2	3	4	5	6	7	8	9	10	11	12	13	14	15	16	17	18	19	20	21	22	23	24	25	26	27	28	29	30	31						
Hap 1	G	A	A	C	C	T	A	G	T	T	C	A	T	T	G	C	C	T	T	T	T	T	G	A	T	T	G	T	T	T	G			1			1
Hap 2	.	G	.	.	.	.	G	.	.	.	.	.	.	.	.	.	.	.	.	.	.	.	A	.	.	.	A	.	.	.	A		1	5	13	14	33
Hap 3	.	G	.	.	.	.	G	.	.	.	.	.	.	.	.	.	.	C	.	.	.	.	A	.	.	.	A	.	.	.	A		1	2			2
Hap 4	A	G	.	.	.	.	G	A	.	.	T	.	.	.	A	.	.	.	C	.	.	.	A	.	C	.	A	.	.	C	A	1	2	1			4
Hap 5	.	G	.	.	.	.	G	T	.	.	.	.	.	.	.	.	.	.	.	.	.	.	A	.	.	.	A	.	.	.	A				1	1	2
Hap 6	A	G	.	.	.	.	G	.	.	.	.	.	.	.	.	.	.	.	.	.	.	.	A	.	.	.	A	.	.	.	A				1	2	3
Hap 7	.	G	.	.	.	.	G	.	.	.	.	.	.	.	.	.	.	.	.	.	.	.	A	G	.	.	A	.	.	.	A					3	3
Hap 8	.	G	.	.	.	.	G	.	.	.	.	.	.	C	.	.	.	.	.	.	.	.	A	G	.	.	A	.	.	.	A					1	1
Hap 9	A	G	.	.	.	.	G	A	.	.	T	.	.	.	A	.	.	.	.	.	.	.	A	.	C	.	A	.	.	C	A		5	10		1	16
Hap 10	.	G	.	.	.	.	G	.	.	.	.	G	.	.	.	.	.	.	.	.	.	.	A	.	.	.	A	.	.	.	A					1	1
Hap 11	.	G	.	.	.	.	G	.	.	.	.	G	.	.	.	.	.	.	.	.	.	.	A	.	.	C	A	.	.	.	A					1	1
Hap 12	.	G	.	.	.	.	G	.	.	.	.	.	.	.	.	.	.	.	.	.	.	.	.	.	.	.	A	.	.	.	A			3		1	4
Hap 13	A	G	.	.	.	.	G	A	.	.	T	.	.	.	A	.	.	.	.	.	.	A	A	.	C	.	A	.	.	C	A		1				1
Hap 14	A	G	.	.	.	.	G	A	.	.	T	.	.	.	A	.	.	.	.	.	.	.	.	.	C	.	A	.	.	C	A		1				1
Hap 15	A	G	.	.	.	.	G	A	.	.	T	.	.	.	.	.	.	.	.	.	.	.	A	.	C	.	A	.	.	C	A	6	2				8
Hap 16	A	G	.	.	.	.	G	A	.	.	T	.	.	.	A	.	.	.	.	.	.	.	A	.	C	.	A	C	C	C	A	1					1
Hap 17	.	.	.	.	.	.	.	.	.	.	.	.	.	.	.	.	.	.	.	.	.	.	A	.	.	.	A	.	.	.	A			2			2
Hap 18	.	G	.	.	.	C	G	.	.	.	.	.	.	.	.	.	.	.	.	.	.	.	A	.	.	.	A	.	.	.	A				1		1
Hap 19	.	G	.	T	T	.	G	.	.	.	.	.	A	A	.	.	.	.	.	A	A	A	A	G	.	.	A	.	.	.	A					1	1
Hap 20	.	G	C	.	.	.	G	.	.	.	.	.	.	.	.	.	.	.	.	.	.	.	A	.	.	.	A	.	.	.	A					1	1
Hap 21	.	G	.	.	.	.	G	.	.	C	.	.	.	.	.	.	.	.	.	.	.	.	A	.	.	.	A	.	.	.	A				1		1
Hap 22	.	G	.	.	.	.	G	.	C	.	.	.	.	.	.	.	.	.	.	.	.	.	A	.	.	.	A	.	.	.	A				1		1
Hap 23	.	G	.	.	.	.	G	.	.	.	.	.	.	.	.	.	.	.	C	.	.	.	A	.	.	.	A	.	.	.	A			1			1
Hap 24	.	G	.	.	.	.	G	.	.	.	.	.	.	.	.	.	T	.	.	.	.	.	A	.	.	.	A	.	.	.	A				1		1
Hap 25	.	G	.	.	.	.	G	.	.	.	.	.	.	.	.	T	.	.	.	.	.	.	A	.	.	.	A	.	.	.	A				1		1

## APPENDIX 2

### *Bagrus docmak* haplotype and corresponding sample source details

**Table:**

Haplotype Name	Sequence Name	Lake Victoria	Victoria Nile River	Lake Albert	Lake Edward	Kazinga Channel	Total number of haplotypes (*n*)
HAP1	ALB_MURC195			1			1
HAP23	ALB_NTK27			1			1
HAP2	ALB_WSK3	0	1	5	13	14	33
HAP3	ALB_WSK4		1	2			3
HAP4	ALB_WSK5	1	2	1			4
HAP17	ALB_WSK9			2			2
HAP21	EDWM4				1		1
HAP18	EDWM5				1		1
HAP22	EDWM7				1		1
HAP25	EDWV19				1		1
HAP5	EDWV6				1	1	2
HAP24	EDWV8				1		1
HAP6	KAZF1				1	2	3
HAP9	KAZF12		5	10		1	16
HAP10	KAZF15					1	1
HAP7	KAZF2					3	3
HAP11	KAZF21					1	1
HAP12	KAZF25			3		1	4
HAP8	KAZF3					1	1
HAP19	KAZF27					1	1
HAP20	KAZF29					1	1
HAP13	NILE_RNK29		1				1
HAP14	NILE_RNK39		1				1
HAP15	NILE_RNK47	6	2				8
HAP16	VIC_RNK7	1					1
Total							93

## APPENDIX 3

### List of microsatellite primers for *Bagrus docmak*

**Table:**

Locus	Primer sequence	*T*_M_ (°C)	Motif	Size range (bp)
Bd04^a^	F: TGTGGACCAAGAGACAGGTG	59	(AGAT)^18^	200–208
	R: AATGAACAAGGCAGGTGATG			
Bd18^a^	F: ATGGGGAGGAAAAGTGGAG	61	(AC)^15^	100–102
	R: CCTGAGTGCATTGCTCATGG			
Bd01^a^	F:TTGCCAATCCTGATGACACTC	60	(TTCT)^15^	203–219
	R:TAAAGCTGGGCAACTGATCC			
Bd02^a^	F:TGTGCTCTGACCCCTACCTC	60	(AGAT)^17^	110–130
	R:GGGTATCGCATCCCAGATAG			
Bd12^a^	F: CCGACCATCTCAAATACAAGTC	60	(AAT)^18^	237–258
	R: CTCTTCCCCAAGGCTATTCC			
Bd09^a^	F: ACTGTTCCCATGAAGTTGGG	60	(ATT)^19^	223–238
	R: TGGTCAACTTTAGATGTGCAGC			
Bd06^a^	F: TTCTGAAGCCCAAAGTAGACG	59	(GATA)^16^	171–199
	R: GCCCACACTATTGACACAGG			
Bd20^a^	F: TCCTGGAGACCAAGACCAAG	60	(CA)^11^	156–168
	R: TGCAGGTTAAGAATGGAGGC			
Bd05^a^	F: GCTGGCAACATGCAGTAATC	59	(ATAC)^15^	136–172
	R: CAGCATTTCATTGCTATGTGC			
BD07	F: GAGCACACGAAACATTGCAG	60	(GATA)^15^	125–157
	R: TTGTAGATTCCCTTTGGGATG			
Bd16	F: GCAATCGCACTCTTGTTATCG	61	(ATT)^13^	83
	R: TAGTAGCGCACCCAGGAAAC			
BD08	F: TTACCTCACACTCTGGGGTTG	60	(ATCT)^16^	179–187
	R: GGTAAAGGTTTACACTGTGGGG			
BD03^a^	F:CCTGCAGGAGTTTGTTTGTG	60	(TAGA)^15^	159–191
	R: CGTGCCATAGGCATTTATCC			
BD14^a^	F: CTTTAATGACACTGCGCTGC	60	(TAT)^17^	218–233
	R: CTCAAAGCGCTTGAAGTGG			
Bd10^a^	F: GTCCCACGGACTGAAAAGTG	60	(TTA)^15^	266–287
	R:TCAACTTCTTAGCACAAAATCAGAC			

*T*_M_, optimal primer melting temperature.

^a^ Polymorphic loci used for analyses in the current study.

Source: Extracted from Basiita et al. (2016), in Microsatellite records by Goossens (2015).
